# Chemogenetic activation of an infralimbic cortex to basolateral amygdala projection promotes resistance to acute social defeat stress

**DOI:** 10.1038/s41598-020-63879-8

**Published:** 2020-04-23

**Authors:** Brooke N. Dulka, Elena D. Bagatelas, Kimberly S. Bress, J. Alex Grizzell, Megan K. Cannon, Conner J. Whitten, Matthew A. Cooper

**Affiliations:** 1 Department of Psychology, University of Tennessee, Knoxville, TN 37996 USA; 2 Present Address: Department of Psychology, University of Wisconsin-Milwaukee, Milwaukee, WI 53211 USA

**Keywords:** Aggression, Neural circuits, Social behaviour, Stress and resilience

## Abstract

Tremendous individual differences exist in stress responsivity and social defeat stress is a key approach for identifying cellular mechanisms of stress susceptibility and resilience. Syrian hamsters show reliable territorial aggression, but after social defeat they exhibit a conditioned defeat (CD) response characterized by increased submission and an absence of aggression in future social interactions. Hamsters that achieve social dominance prior to social defeat exhibit greater defeat-induced neural activity in infralimbic (IL) cortex neurons that project to the basolateral amygdala (BLA) and reduced CD response compared to subordinate hamsters. Here, we hypothesize that chemogenetic activation of an IL-to-BLA neural projection during acute social defeat will reduce the CD response in subordinate hamsters and thereby produce dominant-like behavior. We confirmed that clozapine-N-oxide (CNO) itself did not alter the CD response and validated a dual-virus, Cre-dependent, chemogenetic approach by showing that CNO treatment increased c-Fos expression in the IL and decreased it in the BLA. We found that CNO treatment during social defeat reduced the acquisition of CD in subordinate, but not dominant, hamsters. This project extends our understanding of the neural circuits underlying resistance to acute social stress, which is an important step toward delineating circuit-based approaches for the treatment of stress-related psychopathologies.

## Introduction

Post-traumatic stress disorder (PTSD) is a debilitating illness characterized by exposure to a traumatic event followed by the development of a constellation of symptoms including re-experiencing the event (*e.g*. nightmares or flashbacks), hyperarousal (*e.g*. vigilance or exaggerated startle responses), and avoidance behavior (*e.g*. social withdrawal). Because not all individuals who experience trauma develop PTSD, there has been growing interest in what factors make some individuals resilient to the effects of stress and others susceptible. The amygdala and prefrontal cortex (PFC) are known to regulate emotional responses to aversive stimuli and neural circuitry models have identified these brain regions in the development and expression of PTSD symptoms^1–4^. For example, compared to resilient individuals, those who are PTSD-susceptible display diminished blood oxygen levels in the PFC during an emotion regulation task^5^. One prevailing hypothesis is that variation in PFC and amygdala connectivity underlies stress resilience and emotion regulation^6–8^. Indeed, healthy individuals that are better able to suppress negative emotion during an emotion regulation task show not only greater attenuation of amygdala activity, but also greater inverse coupling between the amygdala and ventromedial PFC (vmPFC)^6^. In addition, exposure to aversive images produces inverse coupling between the vmPFC and amygdala, and vmPFC recruitment upon image onset occurs in a time-dependent manner and predicts stress resilience in situations both inside and outside the laboratory^9^. This inverse coupling is consistent with research from animal models that identifies several mechanisms by which ventral regions of the vmPFC, such as the infralimbic (IL) cortex, inhibit amygdala output^10–16^. Furthermore, pre-existing differences in vmPFC-amygdala connectivity predict susceptibility to the effects of chronic social defeat stress in mice^17^. Also, chemogenetic activation of IL neurons that send projections to the basolateral amygdala (BLA) facilitates the extinction of conditioned fear in mice^18^. Altogether, these findings from humans and rodents suggest that a direct neural projection between vmPFC and amygdala contributes to emotion regulation, fear extinction, and stress resilience.

Social defeat is an ethologically relevant stressor, and acute social defeat has been proposed as a valuable paradigm for investigating neural circuitry controlling behavioral responses to traumatic stress^19,20^. Syrian hamsters show robust territorial aggression, however following acute social defeat, they no longer aggressively defend their home territory in a subsequent social interaction test. Instead, defeated hamsters display submissive and defensive behaviors toward conspecifics, including smaller, non-aggressive intruders. This change in agonistic behavior following acute social defeat stress is called the conditioned defeat (CD) response^21^. We have previously shown that after achieving social dominance, male Syrian hamsters display less submissive and defensive behavior during CD testing when compared to subordinates and animals without a dominance rank (*i.e*. social status controls), which indicates that social dominance promotes resistance to the CD response^22^. Dominant hamsters also show greater defeat-induced c-Fos immunoreactivity (IR) in IL neurons compared to subordinate hamsters^23^. Importantly, pharmacological inactivation of the vmPFC with muscimol reinstates the CD response in dominant hamsters while leaving the CD response of subordinates unchanged, suggesting that vmPFC activity is necessary for resistance to CD in dominants^24^. More recently, we demonstrated that dominant hamsters preferentially activate BLA-projecting IL neurons during acute social defeat stress, while subordinates and social status controls do not^25^. Altogether, these findings suggest that recruitment of BLA-projecting IL neurons during social defeat contributes to CD resistance in dominant hamsters.

In this study, we used a Designer Receptor Exclusively Activated by Designer Drugs (DREADD) strategy to test the hypothesis that chemogenetic activation of an IL-to-BLA pathway during acute social defeat stress would be sufficient to promote a dominant-like CD response in subordinate hamsters. We infused a Cre-dependent, Gq-DREADD virus into the IL and a Cre-expressing retrograde virus into the BLA as a means of selectively targeting IL neurons that send axonal projections to the BLA. We created dominance relationships in pairs of hamsters and then treated animals with clozapine-N-oxide (CNO) during social defeat stress to activate BLA-projecting IL neurons. We predicted that chemogenetic activation of an IL-to-BLA pathway during social defeat would reduce the CD response in subordinate, but not dominant, hamsters.

## Results

### CNO has no effect on conditioned defeat behavior in surgically naïve hamsters

To address possible off-target effects of CNO treatment^26^, we first sought to determine whether CNO alters the CD response in the absence of DREADD virus. In Experiment 1, 36 surgically naïve hamsters received either systemic CNO or vehicle treatment and 30 min later were exposed to acute social defeat stress (CNO, n = 8; vehicle, n = 10) or no defeat control procedures (CNO, n = 8; vehicle, n = 10). All animals were tested for a CD response 24 hours later. During acute social defeat stress, subjects were consecutively placed in the home cages of three separate resident aggressors for 5-min each with 5-min inter-trial intervals, as described previously^27,28^. No defeat control animals were treated identically except that the aggressors were removed from their cages to control for olfactory and environmental exposure.

Systemic CNO treatment in surgically naïve hamsters did not alter the amount of aggression subjects received during social defeat stress, how subjects responded to the aggressor during social defeat, or how the subjects responded in a CD test 24-hours after defeat. There were no significant differences between CNO-treated and vehicle-treated hamsters in the number of attacks received nor the duration of aggression received during social defeat stress (Table 1; t(16) = 0.47, *p* = 0.648, t(16) = 2.08, *p* = 0.054, respectively). We also found that CNO-treated animals (0/8) were not more likely to fight back against aggressors than were vehicle-treated animals (1/10) (χ^2^ = 0.85, *df* = 1, *p* = 0.357).

**Table 1:** Aggression received during acute social defeat.

Experiment – Group	Attacks received (number) (mean ± SEM)	Aggression received (sec) (mean ± SEM)
Exp. 1 – CNO-treated	3.29 ± 0.49	100.87 ± 13.41
Exp. 1 – Vehicle-treated	3.63 ± 0.52	136.79 ± 11.15
Exp. 2 – Functional + CNO	5.52 ± 0.70	177.98 ± 10.87
Exp. 2 – Functional + Vehicle	7.86 ± 1.84	150.60 ± 17.45
Exp. 2 – Nonfunctional + CNO	6.47 ± 0.68	171.85 ± 8.61
Exp. 3 – CNO-treated DOM	5.25 ± 0.47	174.95 ± 7.03
Exp. 3 – Vehicle-treated DOM	5.13 ± 0.54	183.58 ± 9.07
Exp. 3 – CNO-treated SUB	5.28 ± 0.74	166.29 ± 17.94
Exp. 3 – Vehicle-treated SUB	4.50 ± 0.27	122.62 ± 16.87
Exp. 3 – CNO-treated SSC	5.00 ± 0.69	161.78 ± 17.51
Exp. 3 – Vehicle-treated SSC	5.42 ± 0.29	154.11 ± 11.1

Average number of attacks and duration of aggression received per 5 min social defeat (mean ± SEM). n = 5–10 per group. Abbreviations: CNO = clozapine-N-oxide, DOM = dominant, SEM = standard error of the mean, SSC = social status control, SUB = subordinate.

**Figure 1:**
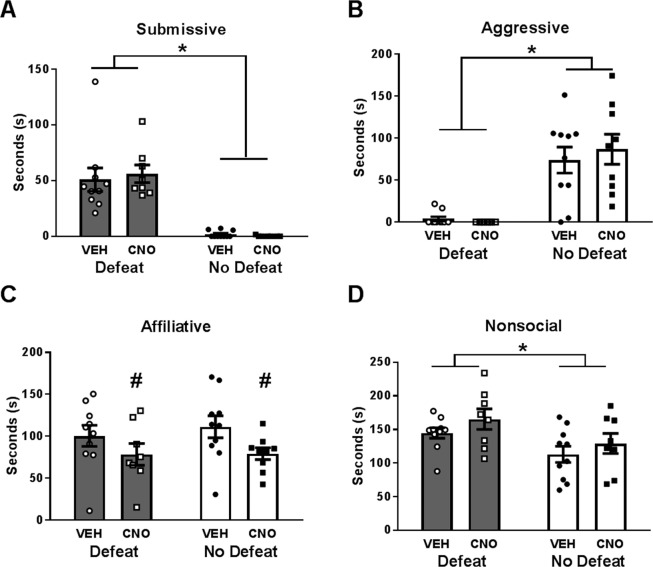
CNO has no effect on CD behavior in surgically naïve hamsters. Durations of (**A**) submissive behavior, (**B**) aggressive behavior, (**C**) affiliative behavior, and (**D**) nonsocial behavior are shown for a 5 min CD test. Data are shown as mean ± SEM. An asterisk denotes a significant main effect of defeat (*p < 0.05), a pound sign denotes a significant main effect of drug (^#^p < 0.05), n = 8–10 per group.

CNO alone also did not affect the CD response. There were no significant drug × defeat interactions in the duration of submissive, aggressive, affiliative, or nonsocial behavior during CD testing (Fig. 1A; *F*_(1, 33)_ = 0.28, *p* = 0.603, Fig. 1B; *F*_(1, 33)_ = 0.47, *p* = 0.496, Fig. 1C; *F*_(1, 33)_ = 0.18, *p* = 0.673, Fig. 1D; *F*_(1, 33)_ = 0.001, *p* = 0.977, respectively). There was also no main effect of CNO treatment on the duration of submissive, aggressive, or nonsocial behavior displayed during CD testing (*F*_(1, 33)_ = 0.07, *p* = 0.792, *F*_(1, 33)_ = 0.14, *p* = 0.711, *F*_(1, 33)_ = 2.76, *p* = 0.106, respectively). On the other hand, CNO treatment significantly decreased the duration of affiliative behavior (*F*_(1, 33)_ = 5.24, *p* = 0.029). As expected, there was a main effect of defeat condition on the duration of submissive, aggressive, and nonsocial behavior (*F*_(1, 33)_ = 62.45, *p* < 0.001, *F*_(1, 33)_ = 40.98, *p* < 0.001, *F*_(1, 33)_ = 6.86, *p* = 0.013, respectively), although not affiliative behavior (*F*_(1, 33)_ = 0.24, *p* = 0.627). Together, these data indicate that acute social defeat affects agonistic behavior in a CD test, but CNO does not.

### A functional dual-virus approach increases c-Fos-IR in the vmPFC

We next validated our chemogenetic approach through immunohistochemistry for the immediate early gene, c-Fos, in the IL. In Experiment 2, 38 animals received either functional or nonfunctional viral vector treatment. Functional viral vector treatment included a Cre-dependent, excitatory DREADD vector injected bilaterally into the IL, and a Cre-expressing, retrograde viral vector injected bilaterally into the BLA. Nonfunctional viral vector treatment included a Cre-dependent, excitatory DREADD vector injected bilaterally into the IL, but no virus injected into the BLA. Three weeks later, animals were exposed to either systemic CNO or vehicle treatment prior to acute social defeat stress (functional virus + CNO, n = 9; functional virus + vehicle, n = 7; nonfunctional virus + CNO, n = 10) or no defeat control procedures (functional virus + CNO, n = 4; functional virus + vehicle, n = 5; nonfunctional virus + CNO, n = 3). Then, animals were sacrificed 60 min after defeat or no defeat exposure, and brain tissue was extracted and processed for c-Fos-IR in the vmPFC and BLA.

We first confirmed that treatment conditions did not alter social defeat experience. In hamsters that received functional virus + CNO, functional virus + vehicle, or nonfunctional virus + CNO, there were no significant differences in the number of attacks received or the duration of aggression received during social defeat stress (Table 1; *F*_(2, 23)_ = 1.14, *p* = 0.337, *F*_(2, 23)_ = 1.72, *p* = 0.201, respectively). No animals fought back against the resident aggressor during any social defeat episodes.

**Figure 2:**
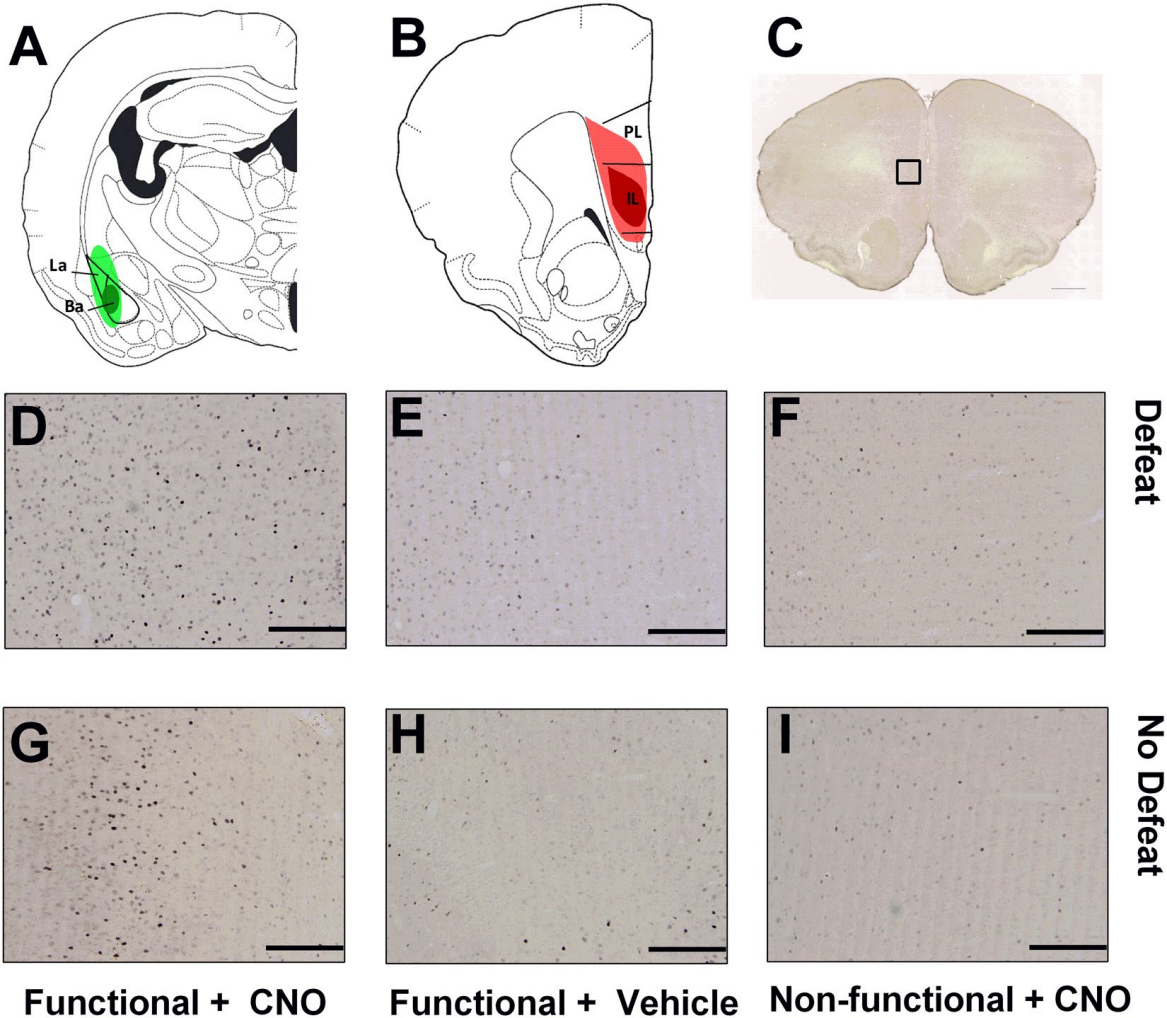
Verification of injection sites and representative c-Fos immunohistochemistry in the vmPFC. (**A**) Reconstruction of the largest (light green) and smallest (dark green) injections of a retrograde Cre vector (CAV2-Cre) in the basolateral amygdala (BLA). (**B**) Reconstruction of the largest (light red) and smallest (dark red) injections of a Cre-dependent hM3D(Gq)-mCherry vector in the infralimbic (IL) and prelimbic (PL) cortex. (**C**) Photomicrograph (2× magnification) of a coronal section of the frontal cortex stitched in a XY plane showing c-Fos IR. Photomicrographs (10× magnification) from representative images of vmPFC c-Fos IR are shown in panels D–I. Images are shown for hamsters that received (**D**) social defeat with functional virus + CNO, (**E**) social defeat with functional virus + vehicle, (**F**) social defeat with nonfunctional virus + CNO, (**G**) no social defeat with functional virus + CNO, (**H**) no social defeat with functional virus + vehicle, and (**I**) no social defeat with nonfunctional virus + CNO. Importantly, there is robust c-Fos-IR in both defeat and no defeat hamsters that received functional virus + CNO treatment. Scale bar in C = 1.0 mm, scale bars in D – I = 200 µm.

**Figure 3:**
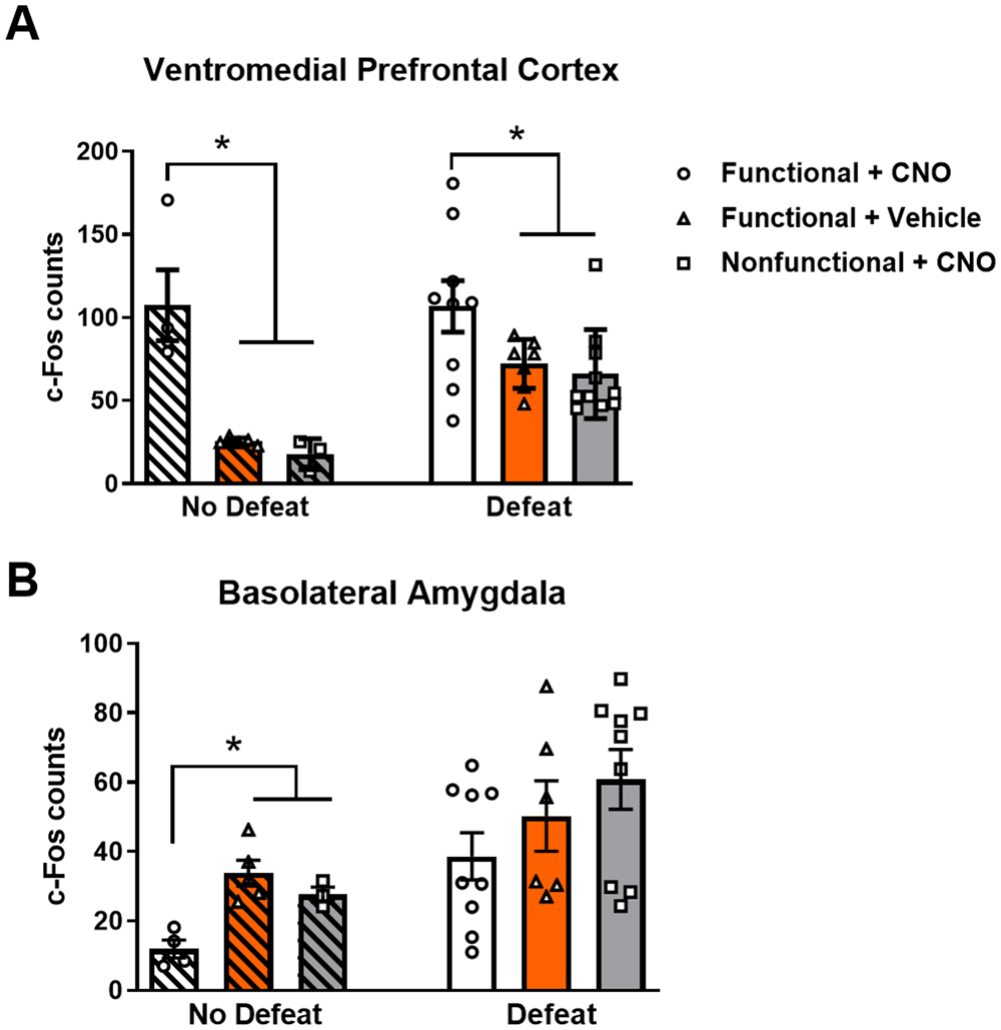
Functional viral vector treatment + CNO is sufficient to increase c-Fos-IR in both defeated and non-defeated hamsters in the vmPFC and decrease c-Fos in non-defeated hamsters in the BLA. (**A**) In non-defeated hamsters, functional + CNO treatment (n = 4) lead to significantly greater c-Fos-IR compared to functional + vehicle (n = 5) and nonfunctional + CNO treatment (n = 3). In defeated hamsters, functional + CNO treatment (n = 9) display significantly greater c-Fos-IR compared to functional + vehicle (n = 7) and nonfunctional + CNO treatment (n = 10). (**B**) Functional + CNO treatment in non-defeated hamsters led to a significant decrease in c-Fos-IR. Data are shown as mean ± SEM. An asterisk indicates a significant difference between groups (*p < 0.05).

A diagram depicting the minimal and maximal spread of Cre-IR in the BLA and mCherry-IR in the IL for this experiment is shown in Fig. 2A,B, respectively. Figure 2C shows a low magnification coronal section of the frontal cortex. Additionally, representative images of c-Fos-IR in the vmPFC are shown in Fig. 2D–I. Importantly, we found significant differences in the expression of vmPFC c-Fos-IR in both defeated and non-defeated animals (Fig. 3A; *F*_(2, 23)_ = 3.99, *p* = 0.032, *F*_(2, 9)_ = 15.32, *p* = 0.001, respectively). Specifically, functional virus + CNO animals that were exposed to acute social defeat stress had significantly greater c-Fos-IR compared to functional virus + vehicle animals and nonfunctional virus + CNO animals exposed to acute social defeat (*p* = 0.050 and *p* = 0.013, respectively). Similarly, functional virus + CNO animals that received no defeat control procedures also had significantly greater c-Fos-IR compared to functional virus + vehicle animals and nonfunctional virus + CNO animals that received no defeat (*p*’s = 0.001). We also confirmed previous research that social defeat increases c-Fos expression in the vmPFC^23^ by comparing all non-defeated and defeated animals that were not assigned to functional virus + CNO treatment groups (*i.e*. pooling nonfunctional and vehicle control groups; t_(32)_ = 5.67, *p* < 0.0001). Together these data show that our dual-virus approach followed by systemic CNO treatment leads to increased c-Fos-IR in the vmPFC, even when c-Fos expression is elevated during social defeat stress. Because IL neurons have been shown to inhibit BLA pyramidal neurons^11^, we also tested whether chemogenetic activation of BLA-projecting vmPFC neurons reduced c-Fos-IR in the BLA (Fig. 3B). We found that non-defeated animals treated with functional virus + CNO showed less c-Fos-IR in the BLA compared to animals treated with functional virus + vehicle and nonfunctional virus + CNO (*F*_(2, 9)_ = 12.93, *p* = 0.002; post-hoc tests *p* = 0.002 and *p* = 0.028, respectively). Although a similar trend was found in defeated animals, the reduction in BLA c-Fos-IR by functional virus + CNO was not statistically significant (*F*_(2, 21)_ = 1.97, *p* = 0.164). To confirm that social defeat increased c-Fos expression in the BLA, we again pooled nonfunctional virus and vehicle control groups and found that defeated animals had more c-Fos-positive cells than non-defeated animals (t_(31)_ = 2.42, *p* = 0.022).

### Selective activation of an IL-to-BLA neural projection is sufficient for resistance to conditioned defeat

**Figure 4:**
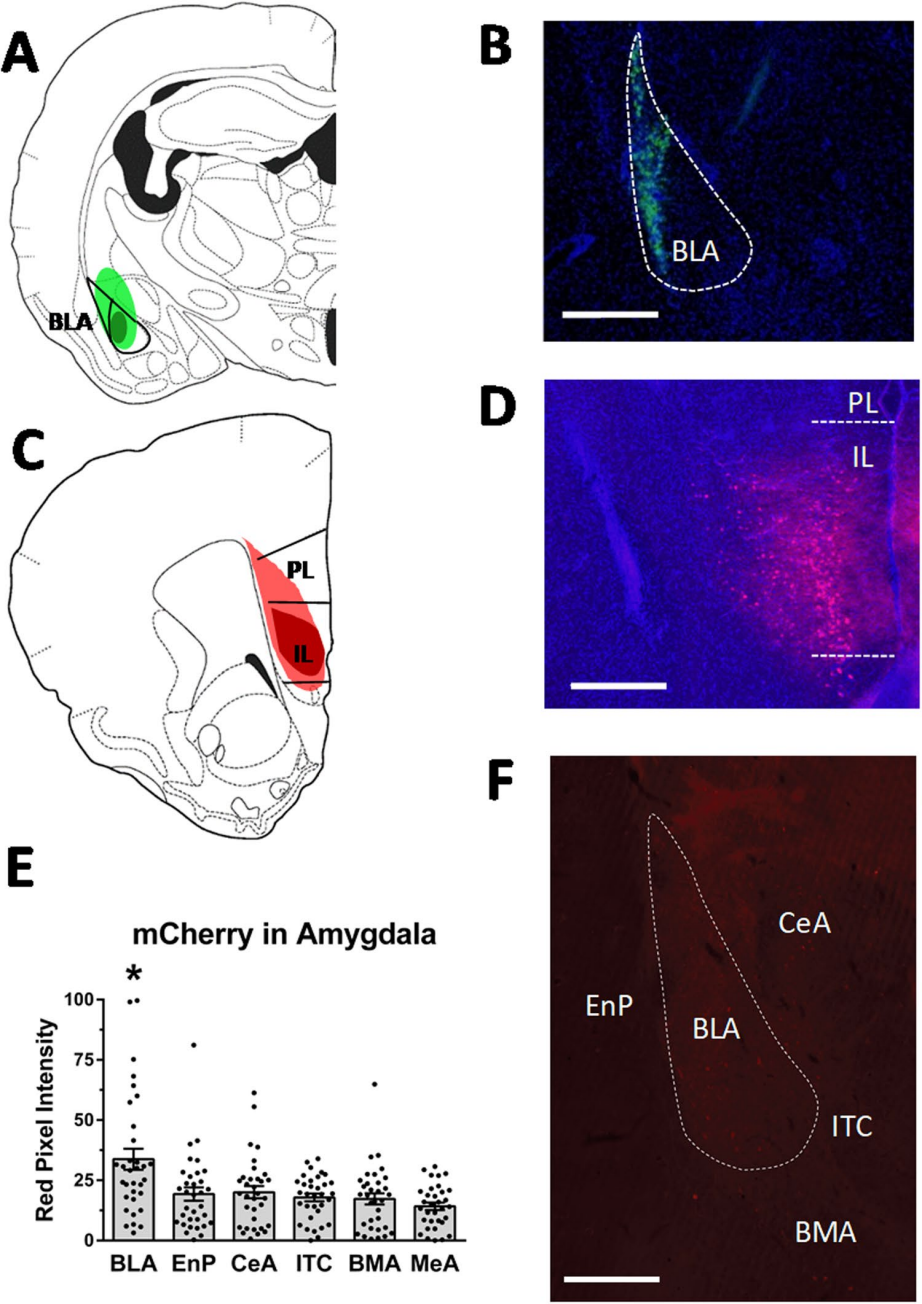
Verification of injection sites and mCherry in the amygdala. (**A**) Reconstruction of the largest (light green) and smallest (dark green) injections of a retrograde Cre vector (rgAAV-pmSyn-EBFP-Cre) in the basolateral amygdala (BLA). (**B**) Photomicrograph (4×) showing representative fluorescence of Cre (green) and DAPI (blue) in the BLA (dotted outline). (**C**) Reconstruction of the largest (light red) and smallest (dark red) injections of a Cre-dependent hM3D(Gq)-mCherry vector in the infralimbic (IL) and prelimbic (PL) cortex. (**D**) Photomicrograph (4× magnification) showing representative fluorescence of mCherry (red) and DAPI (blue) in the IL (dotted lines indicate border of IL). (**E**) We found greater optical density of mCherry-IR in the BLA compared to the endopiriform nucleus (EnP) and other regions of the amygdala. **F**) Photomicrograph captured at 10× and stitched in a XY plane shows that the hM3D(Gq)-mCherry construct travels to nerve endings and can be visualized in the amygdala. Scale bars = 200 µm in (**B**,**D**) and scale bar = 100 µm in (**F**). Abbreviations: BMA = basomedial amygdala, CeA = central amygdala, ITC = intercalated cells.

A diagram depicting the minimal and maximal spread of Cre-IR in the BLA and mCherry-IR in the IL in Experiment 3 is shown in Fig. 4A,C, respectively. Representative images of both Cre-IR and mCherry-IR are shown in Fig. 4B,D, respectively. Histological analysis of mCherry-IR in the vmPFC suggests that retrograde transport of Cre from the BLA enabled expression of the hM3D(G_q_)-mCherry construct primarily in the IL. In addition, the hM3D(G_q_)-mCherry construct travels to nerve endings and can be visualized in the amygdala (Fig. 4F). As expected, we found greater optical density of mCherry-IR in the BLA compared to the endopiriform cortex and other regions of the amygdala (Fig. 4E). Altogether, the histology suggests that our dual-virus approach primarily targeted an IL-to-BLA neuronal projection.

The goal of the final experiment was to determine whether chemogenetic activation of an IL-to-BLA pathway is sufficient to reduce acquisition of CD in subordinate hamsters. In Experiment 3, we injected a Cre-dependent, hM3D(G_q_) viral vector into to IL and a retrograde Cre-expressing virus into the BLA of 44 hamsters, as described above. Animals were paired in daily, dyadic, dominant-subordinate encounters and 24 hours after the final dyadic encounter, dominants (CNO, n = 8; vehicle, n = 8) and subordinates (CNO, n = 6; vehicle, n = 6) received acute social defeat stress. To control for the acquisition and maintenance of social dominance, an additional group of animals remained individually housed and served as social status controls. Social status control animals (CNO, n = 8; vehicle, n = 8) did not receive daily dominance encounters but were exposed to acute social defeat stress. Twenty-four hours after acute social defeat stress, all animals were tested for a CD response. The sample sizes given above reflect animals whose injection sites were localized to both the IL and BLA. Animals with injections sites that were not centralized to the IL and BLA were excluded from analysis.

Dominance relationships were established after 2.18 days of dyadic encounters (SD = 1.12) and dominance status remained consistent throughout the study. First, we tested whether drug treatment or dominance status altered behavior during social defeat stress. There was no significant status × drug interaction (*F*_(2, 38)_ = 1.831, *p* = 0.174) or main effect of drug (*F*_(1, 38)_ = 1.676, *p* = 0.203) in the duration of aggression received during social defeat stress. There was a main effect of status (*F*_(2, 38)_ = 3.340, *p* = 0.046), which indicates that dominants received aggression for a greater duration than others (Table 1). Importantly, there was no significant status × drug interaction (*F*_(2, 38)_ = 0.585, *p* = 0.562), main effect of status (*F*_(2, 38)_ = 0.200, *p* = 0.850) or main effect of drug (*F*_(1, 38)_ = 0.136, *p* = 0.715) in the number of attacks received during social defeat stress (Table 1). We also found that CNO treatment did not alter the proportion of animals that fought back during social defeat in dominants (CNO-treated: 4/8, vehicle-treated: 4/8; χ^2^ = 0.000, *df* = 1, *p* = 1.000), subordinates (CNO-treated: 1/6, vehicle-treated: 0/6; χ^2^ = 1.091, *df* = 1, *p* = 0.296), or social status controls (CNO-treated: 3/8, vehicle-treated: 1/8; χ^2^ = 1.333, *df* = 1, *p* = 0.248). Interestingly, there was a main effect of dominance status on responses to social defeat such that dominant hamsters (8/16) were more likely to fight back than subordinates (1/12) (χ^2^ = 5.458, *df* = 1, *p* = 0.019), but not social status controls (4/16) (χ^2^ = 2.133, *df* = 1, *p* = 0.144).

**Figure 5:**
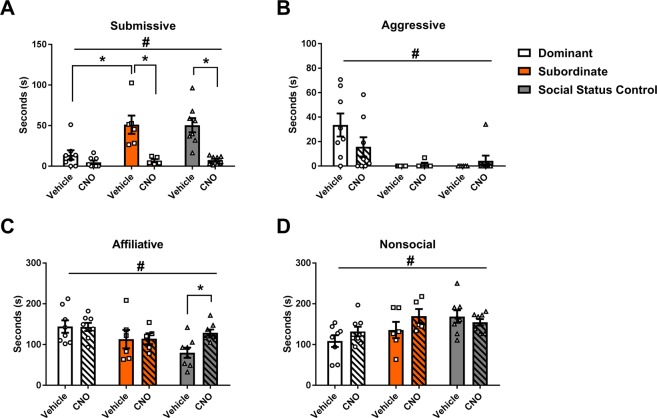
CNO decreases the CD response in subordinates and social status controls. Durations of (**A**) submissive, (**B**) aggressive, (**C**) affiliative, and (**D**) nonsocial behavior are shown during a 5 min CD test. Notably, (**A**) CNO-treated subordinates and social status controls both display a reduced duration of submissive behavior compared to their vehicle-treated counterparts. Vehicle-treated dominants also display a reduced duration of submissive behavior compared to vehicle-treated subordinates. Also, **B**) dominant hamsters show a greater duration of aggressive behavior than other animals. Data are shown as mean ± SEM. An asterisk indicates a significant difference between groups (post-hoc t-test, *p < 0.05). A pound sign indicates a main effect of status (^#^p < 0.05). n = 6–8 per group.

When comparing vehicle-treated dominants and subordinates in the CD test 24 hours later, dominants displayed a reduction in the duration of submissive behavior compared to vehicle-treated subordinates (t_(12)_ = 3.168, *p* = 0.008), which replicates previous findings that dominants have a reduced CD response compared to subordinates^28^. Notably, during CD testing, there was a significant social status × drug interaction in the duration of submissive behavior (Fig. 5A; *F*_(2, 38)_ = 5.283, *p* = 0.009), but not in aggressive, affiliative, or nonsocial behavior (Fig. 5B; *F*_(2, 38)_ = 2.240, *p* = 0.120, Fig. 5C; *F*_(2, 38)_ = 1.909, *p* = 0.162, Fig. 5D; *F*_(2, 38)_ = 1.177, *p* = 0.319, respectively). In planned comparisons, vehicle-treated subordinates showed a significantly greater duration of submissive behavior compared to CNO-treated subordinates (t_(10)_ = 3.759, *p* = 0.004). Similarly, vehicle-treated social status controls showed a significantly greater duration of submissive behavior compared to CNO-treated social status controls (t(14) = 4.814, *p* < 0.001). On the other hand, vehicle-treated dominants did not statistically differ from CNO-treated dominants (t_(14)_ = 1.449, *p* = 0.169), which may be due to a floor effect on total submissive behavior. In addition, CNO-treated social status controls displayed significantly higher affiliative behavior compared to their vehicle-treated counterparts (t_(14)_ = 3.344, *p* = 0.005). These findings indicate that CNO treatment reduced the CD response in subordinates and status social controls, but not in dominants. Furthermore, there was a main effect of social status in the duration of aggressive, affiliative, and nonsocial behavior (*F*_(2, 38)_ = 10.996, *p* < 0.001; *F*_(2, 38)_ = 4.432, *p* = 0.019; and *F*_(2, 38)_ = 4.656, *p* = 0.016, respectively). Altogether, these findings indicate that dominant animals showed more aggression and affiliation and less nonsocial behavior compared to subordinates and social status controls and that CNO treatment produced a dominant-like CD response in subordinates and social status controls.

These findings cannot be explained by the residency status of the dominant and subordinate individuals. When animals were analyzed as residents and intruders, rather than dominants and subordinates, there was no significant residency status × drug interaction in the duration of submissive behavior (*F*_(1, 24)_ = 1.442, *p* = 0.242). There was also no main effect of residency status (*F*_(1, 24)_ = 1.751, *p* = 0.198), but, importantly, the main effect of CNO remained significant (*F*_(1, 24)_ = 10.266, *p* = 0.004).

## Discussion

The present study was designed to elucidate the role of an IL-to-BLA neural projection in stress resilience. Here, we demonstrate that chemogenetic activation of IL-to-BLA neurons during social defeat stress is sufficient to produce resistance to CD in both subordinates and social status controls. Additionally, we demonstrate that selective activation of IL neurons that project to the BLA increases c-Fos expression within the vmPFC and decreases c-Fos expression within the BLA. This study extends our previous findings that vmPFC activity during social defeat is necessary for resistance to CD in dominant hamsters^24^ by demonstrating that selective activation of BLA-projecting IL neurons during social defeat is sufficient to promote resistance to CD in those that had not achieved social dominance. In addition, these findings are consistent with previous research showing that dominant hamsters display increased activation of an IL-to-BLA projection following social defeat stress compared to subordinates and social status controls^25^. The present study builds upon this line of research by demonstrating a causal role for activity within an IL-to-BLA neural projection in the acquisition of CD resistance.

Given recent concerns about the off-target effects of CNO and its potential conversion to clozapine^29^, it was important to ensure that CNO had no effect on the CD response in surgically naïve hamsters. For example, a relatively low dose of CNO (1 mg/kg) decreases the startle response to loud acoustic stimuli, while a higher dose (5 mg/kg) significantly attenuates amphetamine-induced hyperlocomotion^26^. Here, we show that CNO treatment (3 mg/kg) in surgically naïve animals reduced affiliative behavior but did not alter submissive or aggressive behavior, which indicates that CNO treatment itself does not alter the agonistic behavior central to the conditioned defeat response. Furthermore, CNO treatment did not alter c-Fos expression in the vmPFC or BLA in animals without functional virus treatment.

Next, we validated a dual-virus, DREADD approach through c-Fos immunohistochemical analysis. In short, a functional DREADD vector combined with CNO treatment increased c-Fos-IR in the vmPFC, regardless of social defeat exposure. Interestingly, social defeat did not maximize c-Fos expression in the vmPFC, as the number of c-Fos-IR cells was further increased with CNO. However, there did appear to be a ceiling effect on CNO-induced c-Fos expression in the vmPFC, as defeated and non-defeated animals showed a similarly high number of c-Fos-IR cells following CNO treatment. Furthermore, we found that CNO and DREADD treatment reduced c-Fos-IR in the BLA, which is consistent with the view that activation of an IL-to-BLA pathway leads to feedforward inhibition of BLA pyramidal neurons^11^. Interestingly, chemogenetic activation of an IL-BLA pathway did not significantly reduce BLA c-Fos-IR in defeated animals. One possibility for this inconsistency is that acute social defeat increases c-Fos expression in multiple BLA cell types, which increases noise and makes a chemogenetic-induced decrease in c-Fos-IR more difficult to detect. For example, restraint stress has been shown to increase c-Fos expression in parvalbumin (PV), calbindin, and calcium/calmodulin-dependent kinase II-positive neurons in the BLA^29^. Similarly, anxiogenic drugs increase c-Fos expression in both PV + and PV- cells in the BLA^30^. These studies indicate that acute stress activates a heterogeneous population of cells in the BLA, which might partially mask the effect of activity in IL afferents. Ultimately, our study suggests that reduction of BLA c-Fos-IR is feasible following chemogenetic activation of IL-BLA neurons, although we likely lack statistical power to detect significant BLA c-Fos changes during social defeat stress.

In our final experiment, we show that chemogenetic activation of BLA-projecting IL neurons during social defeat stress was sufficient to promote CD resistance in subordinate hamsters and controls without a dominance status. In addition, CNO treatment increased affiliative behavior in control hamsters without a dominance status, which is consistent with a reduction in the CD response. These findings indicate that activation of an IL-to-BLA pathway during social defeat reduces the CD response in animals that do not ordinarily activate this pathway, although it has no demonstrable effect in dominant animals who activate this pathway endogenously. This study cannot address whether CNO treatment recruits an IL-to-BLA pathway in subordinate hamsters to a similar extent expected in dominants or whether it produces supraphysiological activation. Similarly, it is unknown whether CNO treatment can appreciably increase vmPFC activity in dominant hamsters. Regardless, the low submissive behavior in vehicle-treated dominant hamsters creates a floor effect on the CD response, which limits our ability to detect a reduction of CD brought on by chemogenetic activation of an IL-to-BLA pathway in dominants. Interestingly, we found that CNO treatment reduces the CD response in subordinate hamsters without altering fighting back against the trained aggressor during social defeat stress. This finding suggests that chemogenetic activation of an IL-to-BLA pathway contributes to CD resistance without modulating how animals respond to the social defeat experience itself.

We know broadly that neural activity in the vmPFC is both necessary and sufficient for the acquisition, but not expression, of the CD response^31^. However, the function of specific vmPFC efferent projections in acute social defeat is not yet known and the role of IL and prelimbic (PL) regions of the vmPFC has not been delineated for CD. To the best of our knowledge, this is the first study to elucidate a role for an IL-to-BLA neural projection in resistance to acute social defeat stress. This finding is consistent with previous research using a mouse model of chronic social defeat stress^32,33^, although multiple pathways likely contribute to stress resilience in mice and hamsters^34,35^.

While an IL-to-BLA pathway is commonly associated with fear extinction^10,18,36,37^, our findings suggest this pathway contributes to status-dependent differences in the acquisition of stress-induced behavioral changes and highlight a more expansive role for BLA-projecting IL neurons. The defeat-induced changes in behavior that characterize the CD response in Syrian hamsters also require a well-delineated set of neurochemical signals in the BLA. For example, pharmacological blockade of the NR2B subunit of the N-methyl-D-aspartate (NMDA) receptor in the BLA reduces the acquisition, but not expression, of CD^38^. The overexpression of cAMP response element binding (CREB) protein in the BLA enhances the acquisition of CD^39^. Activation of tyrosine kinase B (TrkB) receptors in the BLA is also required for the acquisition of the CD response^40^. Importantly, the acquisition and expression of associative learning during Pavlovian fear conditioning requires a similar set of neurochemical signals in the BLA^41–45^. Future research will be necessary to determine whether activation of an IL-to-BLA circuit blocks specific cellular mechanisms in the BLA that support the formation of the CD response. It is also important to keep in mind that other projections from the vmPFC play a key role in behavioral responses to stress. For instance, the vmPFC detects stressor controllability via reciprocal connections with the dorsomedial striatum and then inhibits stress-induced activation of the serotonin system via projections to the dorsal raphe nucleus^46,47^.

A dual-virus chemogenetics approach is not without limitations. For instance, the hM3D(Gq) injections occasionally spread dorsally into the PL. Inasmuch, it is possible that some PL projections to the BLA may have contributed to changes in the acquisition of the CD response. While the PL and IL have separate roles in the expression and extinction of conditioned fear in rats^37^, others have shown that PL and IL neurons produce similar feedforward inhibition in BLA pyramidal neurons^11^. Also, we have previously shown that social defeat increases neural activity in both BLA-projecting IL and PL neurons in dominant hamsters^25^. Thus, IL and PL projections to the BLA may function similarly in acutely defeated hamsters. Separately, while injections of retrograde Cre vectors were centralized to the BLA, cells medial and lateral to the BLA were moderately transfected with Cre in some animals. The GABAergic intercalated (ITC) cells medial to the BLA provide feed-forward inhibition to the central amygdala (CeA) in response to glutamatergic input into the BLA^48^. Importantly, the IL sends robust projections to these cells^49^, and these ITC cells are required for the expression of fear extinction^14^. However, the ITC cell populations are too small to target with microinjections and other genetic or electrophysiological approaches will be needed to further investigate an IL-to-ITC neural projection in dominant and subordinate animals. Similarly, the endopiriform cortex lateral to the BLA also displayed moderate amounts of Cre-IR in some animals, and this region also receives input from the IL^50^. While the piriform cortex appears to be involved in the integration of odor representations^51^, the role of an IL-to-endopiriform cortex remains undefined. While we cannot rule out a role in stress resilience for IL projections to ITC cells or endopiriform cortex, as well as potential axonal collaterals outside the amygdala, our data indicate that the primary target of our dual-virus approach was IL neurons with terminals in the BLA. Interestingly, we found a relatively small number of mCherry-positive cell bodies in the BLA, which suggests low level transynaptic transport of AAV8. This observation was surprising because AAV8 has been reported to exhibit very low transynaptic expression^52^, although the expression patterns of AAV vectors vary by species, brain region, and cell type. The existence of mCherry-positive cells in the BLA raises the possibility that CNO treatment may have stimulated local inhibitory circuits within the BLA as well as activated IL afferents. While our approach cannot distinguish between these possibilities, they are both consistent with a key role for an IL-BLA pathway in reducing the CD response. However, as an additional caveat, the role of non-DREADD expressing cells in the vmPFC should be considered. Previous work indicates that CNO treatment can reduce c-Fos expression in both DREADD-positive and DREADD-negative cells in the prefrontal cortex^53,54^. Thus, future work is needed to address whether systemic CNO treatment activates DREADD-positive cells only and whether neighboring non-DREADD cells can modulate the effects of systemic CNO treatment.

In summary, the results of the current study support the view that selective activation of an IL-BLA pathway during social defeat stress reduces the acquisition of the CD response in animals that do not ordinarily activate this pathway. While dominant hamsters appear to activate this neural circuit naturally^25^, resistance to CD can be instilled in subordinates and in animals without social dominance by artificially activating this circuit. This project extends our understanding of the neural circuits underlying resistance to social stress, which is an important step towards delineating a circuit-based approach for the prevention and treatment of stress-related psychopathology.

## Methods

### Subjects

Subjects were adult male Syrian hamsters (*Mesocricetus auratus*) obtained from our breeding colony that was derived from animals purchased from Charles River Laboratories. Subjects were approximately 3 months old (120–180 g) at the start of the study. Older hamsters (>6 months,>190 g) were individually housed and used as resident aggressors for social defeat stress. Younger hamsters (~2 months, <120 g) were housed in groups of four and used as non-aggressive intruders for conditioned defeat testing. All animals were housed in polycarbonate cages (12 cm × 27 cm × 16 cm) with corncob bedding, cotton nesting materials, and wire mesh tops. Food and water were available *ad libitum*. Subjects were handled several times the week before behavioral procedures occurred to habituate them to the stress of human handling. Animals were housed in a temperature-controlled colony room (21 ± 2 °C) and kept on a 14:10 h light:dark cycle to maintain reproductive condition. All behavioral tests were performed during the first 3 h of the dark phase of their cycle, which coincides with a circadian peak in aggressive behavior. All procedures were approved by the University of Tennessee Institutional Animal Care and Use Committee and are in accordance with the National Institutes of Health Guide for the Care and Use of Laboratory Animals.

### Stereotaxic surgery and viral infusions

Animals were anesthetized with isoflurane, mounted in a stereotaxic instrument, and received either a functional (Experiments 2 and 3) or nonfunctional (Experiment 2 only) viral vector treatment. Functional viral vector treatment included a Cre-dependent, excitatory DREADD vector injected bilaterally into the IL, and a Cre-expressing, retrograde viral vector injected bilaterally into the BLA. Nonfunctional viral vector treatment included a Cre-dependent, excitatory DREADD vector injected bilaterally into the IL but no virus injected into the BLA. All viral microinjections occurred at a rate of 0.05 µl/min for 10 min, resulting in a total injection volume of 0.5 µl/side, and the microsyringe was left in place for 10 min to allow for diffusion. For BLA injections, the stereotaxic coordinates were 0.6 mm posterior and 3.95 mm lateral to bregma and 6.5 mm below dura. Injections into the IL occurred at a 20° angle toward the midline and the stereotaxic coordinates were 3.7 mm anterior to bregma, 1.65 mm lateral to bregma, and 4.5 mm below dura. Animals received their surgeries such that viral vector infusion occurred 3 weeks prior to acute social defeat stress.

The Cre-dependent, excitatory DREADD was an AAV8-hSyn-DIO-hM3D(G_q_)-mCherry vector (4.8 × 10^12^ GC/ml titer) contributed by Bryan Roth (Addgene viral prep #44361-AAV8)^55^. The Cre-expressing, retrograde viral vector was either a canine adenovirus type 2 (CAV2-Cre; 4.1 ×10^12^ pp/ml titer; Experiment 2) contributed by Eric Kremer from the Institut de Génétique Moléculaire de Montpellier^56^ or a rgAAV-pmSyn-EBFP-Cre vector (7.6 × 10^12^ GC/ml titer; Experiment 3) contributed by Hongkui Zeng (Addgene viral prep #51505-AAVrg)^57^.

### Dominant-subordinate encounters

To allow the establishment of dominance relationships, subjects were individually housed for 1 week following stereotaxic surgery and were then weight-matched into resident-intruder dyads and paired in daily social encounters for 14 days as described previously^27,28^. Briefly, subjects were randomly assigned as a resident or intruder, and all social encounters occurred in the resident’s home cage. Encounters were 10 min in duration prior to the establishment of dominance relationships, while all subsequent encounters were 5 min. Dominant and subordinate animals were identified by the direction of agonistic behavior within each dyad. If a dyad did not form a dominance relationship after 5 encounters, both animals from that dyad became social status controls. Other social status controls were never paired in dominance interactions and they did not significantly differ from unformed dyads on any measure.

### Acute social defeat stress and drug injections

Animals received an injection of either CNO (Hello Bio: HB1807) or vehicle 30 min prior to acute social defeat stress. CNO was dissolved in 5% dimethyl sulfoxide (DMSO) and saline to a concentration of 1.6 mg/ml. CNO or vehicle (5% DMSO in saline) was administered by intraperitoneal injection to each hamster (3 mg/kg; 0.3 ml volume injection). Acute social defeat stress consisted of subjects being placed in the home cages of three separate resident aggressors, as described previously^27,28^. Briefly, resident aggressors were prescreened to ensure that they reliably attacked and defeated intruders. Subjects were exposed to three resident aggressors in consecutive 5-min aggressive encounters at 5-min inter-trial intervals. The first defeat episode began when the subject submitted to an attack from the resident aggressor. Subjects submitted immediately in the second and third defeat episodes. No defeat control animals were placed in the empty home cages of three separate resident aggressors for three 5-min exposures to control for the novel environment and olfactory cues associated with social defeat stress. Social defeat encounters were digitally recorded for behavioral analysis. The number of attacks received during social defeat stress, the duration of aggressive behavior received during social defeat stress, and whether or not subjects fought back against the resident aggressor during the first defeat were scored by a single observer blind to treatment conditions. The observer achieved 90% agreement on an ethogram of aggressive behavior using existing video files dedicated for reliability training. Aggressive encounters were carefully monitored for wounding and animals that received minor scratches were treated with antiseptic solution. No animal received a wound that resulted in signs of pain or distress and none of the animals were removed from the study because of wounding.

### Conditioned defeat testing

CD testing was conducted as described previously^28^. Briefly, CD testing consisted of a 5 min social interaction test, during which a non-aggressive intruder was placed in the subject’s home cage. Non-aggressive intruders were younger, group-housed animals that displayed social and nonsocial behavior, and we excluded those intruders that displayed aggressive behavior. All testing sessions were digitally recorded with Sony low light camcorders and the behavior of the subject was quantified using behavioral analysis software (Noldus Observer). We quantified the total duration of submissive/defensive behavior (flee, avoid, upright and side defensive postures, tail-up, stretch-attend, head flag); aggressive behavior (chase, attack including bite, upright and side offensive postures); affiliative behavior (nose touching, sniff, approach); and nonsocial behavior (locomotion, grooming, nesting, feeding). We also quantified the frequency of flees and attacks displayed by the subject. Behavioral quantification was performed blind to treatment conditions, and inter-rater reliability was established on a subset of videos by reaching 90% agreement on the duration of submissive/defensive and aggressive behavior.

### Histology and immunohistochemistry

In Experiment 2, 60 min after acute social defeat stress or no defeat control procedures, all animals were anesthetized with isoflurane and transcardially perfused with 100 ml of 0.1 M phosphate buffered solution (PB; pH 7.4) followed by 100 ml of 4% paraformaldehyde. Brains were removed and post-fixed in 4% paraformaldehyde for 24 h, followed by 0.1 M PB/30% sucrose solution for 48 h, and then were stored in cryoprotectant at 4 °C. A consecutive series of 40 µm coronal sections were cut while submerged in PB on a vibrating microtome, and the prefrontal cortex and amygdala were collected separately and stored as free-floating sections in cryoprotectant at 4 °C. In Experiment 3, brain tissue was collected following CD testing and similarly processed.

For c-Fos labeling, sections were rinsed before each incubation in five 10 min washes with a phosphate buffered Triton solution (PB-Tx; 0.2% Triton X-100 in 0.1 M PB, pH 7.4), conducted at room temperature (RT). Sections were quenched for endogenous peroxidase activity in 0.3% hydrogen peroxide and 30% methanol solution for 25 min. Sections were then incubated in 1% goat serum (GS) in PB-Tx for 25 min to block non-specific binding. Next, sections were incubated overnight at RT in c-Fos primary antibody in PB-Tx at 1:5,000 concentration (rabbit anti-c-Fos, Santa Cruz: sc-52). Following incubation in the c-Fos primary antibody solution, the sections were then incubated for 1 hr in PB-Tx with 1% GS and biotinylated goat, anti-rabbit IgG antibody at 1:200 concentration (Vector Laboratories: BA-1000). Sections were next incubated for 1 hr in PB-Tx with an avidin-biotin complex (ABC Kit, Vector Laboratories: PK-4000), and the peroxidase reaction was visualized using a 10 min incubation in 50% 3,3’-diaminobenzidine (DAB tablet, Sigma-Aldrich: D5905) with ammonium nickel sulfate hexahydrate and hydrogen peroxide dissolved in PB. After immunohistochemistry, sections were washed with distilled H_2_O prior to being mounted on glass microscope slides. After air-drying for 48 hrs, sections were dehydrated using a series of alcohols, cleared with citrosolv, and coverslipped using Permount (Fisher Scientific). Prefrontal cortex and amygdala tissue was processed separately for c-Fos labeling.

For mCherry labeling, sections were first quenched for endogenous peroxidase activity in 0.3% hydrogen peroxide in PB-Tx for 25 min. Sections were then incubated in 3% donkey serum (DS) in PB-Tx for 25 min to block non-specific binding. Next, sections were incubated overnight at RT in a mCherry primary antibody in PB-Tx at 1:2,500 concentration (dsRed made in rabbit, Clontech: 632496). Sections were then incubated for 1 hr in PB-Tx with 1% DS and Alexa Flour 594, donkey anti-rabbit IgG antibody at 1:200 concentration (ThermoFisher: R37119). From this step forward, sections were protected from light. Sections were then rinsed in two 10 min PB washes and washed briefly with distilled H_2_O and mounted on glass microscope slides. After air-drying for 10–30 minutes, a Vectashield containing 4′,6′-diamidino-2-phenylindole dihydrochloride (DAPI) counterstain (Vector: H-1200) was applied, and the slides were coverslipped and sealed with several drops of clear nail polish before storing flat at 4 °C.

For Cre labeling, sections were incubated in 10% GS for two hours to block nonspecific binding. After washing once in PB for 5 min, sections were incubated overnight at 4 °C in a Cre primary antibody in 0.5% PB-Tx (PB-Tx; 0.5% Triton X-100 in 0.1 M PB, pH 7.4) with 2% GS at 1:1,000 concentration (mouse anti-Cre, Millipore: MAB3120). Sections were then incubated for 2 hr in 0.5% PB-Tx with 2% GS with biotinylated goat, anti-mouse IgG antibody at 1:500 concentration (Vector: BA-9200). Next, sections were incubated in a Streptavidin Alexa Fluor 488 conjugate at 1:250 concentration (Life Technologies: S32354). Sections were then rinsed in two 10 min PB washes and processed as described above for mCherry staining.

### Immunohistochemical quantification

For c-Fos immunohistochemistry, images were captured at 10× magnification using an Olympus BX51 microscope. vmPFC images were collected from an 870 μm × 660 μm region that spanned the border of the PL and IL. BLA images were captured from an 870 μm × 660 μm region that was adjacent to the Cre virus injection site. The number of c-Fos immunopositive cells were determined in the vmPFC and BLA using MCID Core image analysis software (InterFocus Imaging). For each image, we recorded background IR in unstained regions of tissue. We then defined immunopositive cells as those that showed staining 1.75× (BLA) or 1.8× (vmPFC) darker than background optical density calculated for each image. MCID software thresholds were calibrated to yield cell counts that were consistent with manual quantification. For each animal, we quantified three to six images along a rostral-caudal axis, and an individual blind to treatment condition collected images and performed quantifications. For mCherry and Cre immunohistochemistry, the injection sites were verified by an observer blind to treatment conditions. Animals were excluded from analyses if mCherry-IR and Cre-IR were not both centralized in the IL and BLA, respectively. For some animals, Cre-IR spread medial and lateral to the BLA into the ITC and endopiriform cortex, respectively. Also, mCherry-IR spread into the PL in some animals. For analysis of mCherry terminals, we identified the amygdala section with the greatest mCherry expression and captured a single image from the BLA, CeA, ITC, basomedial amygdala, medial amygdala, and endopiriform nucleus. We used MCID software to calculate mean red pixel intensity, which reflects the optical density of red pixels within the image.

### Statistical analysis

In Experiment 1, we performed a two-way ANOVA to investigate main effects and an interaction between defeat (2 levels) and drug treatment (2 levels). In Experiment 2, we performed one-way ANOVAs followed by LSD post hoc tests to investigate the effects of CNO and viral vector treatment on c-Fos-IR. In Experiment 3, we performed two-way ANOVAs to investigate an interaction between social status (3 levels) and drug treatment (2 levels) on behavior at CD testing followed by t-tests for specific planned comparisons. We also performed t-tests as a planned comparison to test whether social defeat increased c-Fos-IR in Experiment 2 and whether vehicle-treated dominants differed from vehicle-treated subordinates in Experiment 3. In all three experiments, we performed either a t-test, one-way ANOVA, or two-way ANOVA, where appropriate, to investigate differences in the duration of aggression and number of attacks received during social defeat stress. We also used chi-square tests to analyze the proportion of animals that fought back against the resident aggressor during social defeat. All statistical tests were two-tailed, and α was set at p < 0.05.

## Data Availability

The datasets generated during the current study are available from the corresponding author on reasonable request.
